# Books Review

**Published:** 1870-01

**Authors:** 


### Books Review.

### Transactions of the American Medical Association, Vol. XX., 1869.

A good idea of the scope and value of this Volume will be gained by noticing the following, which appears as the "Table of Contents:"

MINUTES OF THE TWENTIETH ANNUAL MEETING OF THE AMERICAN MEDICAL ASSOCIATION.

Report of the Committee on Publication.

Report of the Treasurer.

Address of William O. Baldwin, M. D., President of the Association.

Report of the Committee on Medical Ethics.

Report of the Delegate of the British Medical Association.

Letter to Professor Ehrenberg, of Berlin.

Report of the Committee on Plan for Relief of Widows and Orphans of Medical Men.

Report of the Committee on Specialties, and on the Propriety of Specialists Advertising.

Report on the Practicability of Establishing a Library of American Medical Works.

Report of the Committee on Medical Education.

Minutes of the Section on the Practice of Medicine in obstetrics.

Report of the Committee on the Training of Nurses.

A Peculiar Appearance of the Tongue in Malarious Diseases. By Thomas C. Osborn, M. D. of Greensboro'. Alabama.

The Prophylactic and the Therapeutic Uses and Abuses of Quinia and its Salts: A Revised Essay. By Stephen Rogers, M. D.

Report of the Committee on the Relations of Alcohol to Medicine. By John Bell, M. D., Chairman.

On Warm Cerebro-Spinal Bath in Congenital Apnœa, and on a New Method of Artificial Respiration. By E. D. McDaniel, A. M., M. D., of Camden, Alabama.

Minutes of the Section on Anatomy and Surgery.

Mollities Ossium (Malakosteon, Osteo-Malacia, Osteo-Sarcosis, Knochen-Erweichung, Rachitismus Adultorum. Rickets, or Softening of the Bones in the Adult). By Joseph Jones, M. D.

A Canula Needle for Applying Ligatures and Stitches. By Paul F. Eve, M, D., of Nashville, Tennessee.

Report of the Best Methods of Treatment for Different Forms of Cleft Palate. By Wm. R. Whitehead, M. D., of New York.

Remarks upon some Points Referring to Success in the Operation of Vesico-Vaginal Fistula. By. M. Schuppert, M. D., of New Orleans.

Minutes on the Section on Meteorology, Medical Topography, and Epidemic Diseases.

On the Cryptogamic Origin of Disease, with Special Reference to Recent Microscopic Investigations on the Subject. By Edward Curtis, M. D.

Report of the Committee on Climatology and Epidemics for the State of New York. By William Faulds Thoms, M. D., Chairman.

Report on Prophylactics in Zymotic Diseases. By Nelson L. North, M. D., of Brooklyn, N. Y.

Report on the Climatology and Epidemic Diseases of Texas for 1868. By T. J. Heard, M. D., of Galveston, Texas.

Report on the Epidemics of California in 1868. By F. W. Hatch, M. D., of Sacramento, California.

Report of the Committee on Climatology and Epidemics in Massachusetts, 1868–69. By Henry I. Bowditch, M. D.

Minutes of the Section on Medical Jurisprudence, Hygiene and Philosophy.

Three Cases of Lead Palsy from the Use of the Cosmetic called "Laird's Bloom of Youth." By Lewis A. Sayre, M. D.

Report of the American Medical Association, on the Subject of the Appointment of a Commissioner in each Judicial District or Circuit, to Aid in the Examination of Witnesses in every Trial involving Medico-Legal Testimony. John Ordronaux, M. D.

Report to the Delegate to the Association of Superintendents of Asylums for the Insane for 1867–68.

PRIZE ESSAY—Quinine as a Therapeutic Agent. By Dr. S. S. Herrick, of Louisiana.

PRIZE ESSAY.—The Physiological Effect and Therapeutic uses of Atropia and its Salts. By Roberts Bartholow, M. D., of Ohio.

Observations and Researches on Albinism in the Negro Race. By Joseph Jones, M. D.

Plan of Organization for a National Medical Association.

Code of Ethics of the American Medical Association.

OFFICERS AND PERMANENT MEMBERS.

Catalogue of the Officers of the American Medical Association.

Permanent Members.

Many of these Papers and Reports are of great value and interest, and where all are so well chosen and instructive it is difficult to select any one for particnlar notice. The Volume as a whole fully sustains the reputation of the Association as the representative body of the profession in the United States, while the Reports and Papers generally reflect great credit upon their authors, as scientific, progressive and earnest men. Some of the papers embodied in this work, have been received in pamphlet form, and will come up for consideration as opportunity offers.

Our space will not permit of anything like a resume of the reports and papers, and we can only assure our readers that the volume of the Transactions of the American Medical Association should be in the hands of every physician. Indeed, it would seem that such assurance would be quite unnecessary, and that every member of the profession would make sure of obtaining a copy upon the first possible opportunity. The work shows the efficiency of the Permanent Secretary,

Wm. B. Atkinson, M. D., who moulds and finishes with great skill whatever he touches.

---

*The American Journal of Syphilography and Dermatology.* By M. M. Henry, M. D.

The editor in commencing the publication of this journal shows an energy and courage worthy admiration, and a hearty support to an American journal of this character will be a credit to the American profession, and especially so since the failure of a similar English publication. The diagnosis of many forms of venereal and cutaneous disease is to the accomplished hospital physician at times quite difficult; much more difficult also, must it be to the practitioner who is isolated from opportunities for special study, to decide in these cases as they are presented to him. The introductory statement of the aims and scope of the journal says that it "can only regard specialties and general practice as mutually supportive, and believes he is the best specialist who does not suffer himself to lose sight of the general current of medical science; and as a corollary, that he is the best general practitioner who keeps close watch of the labors of special investigators.

---

*The Medical Adviser. A Treatise on the Theory and Practice of Medicine, especially adapted to Family Use.* By Rezin Thompson, M. D. Chicago, 1870.

We have received from the publisher specimen sheets of this work, from which we see that the work (which is by the author of a work on "Fevers, Dysentery, etc.) treats concisely, intelligently and correctly upon the topics it presents, and the accompanying illustrations are frequent and remarkably good.

---

*Last Illness of Dr. Alden March. A Criticism on the Management of the Case.* By Charles A. Robertson, A. M., M. D.

This is truly a critical review of the report of the attending physicians made to the friends or Prof. March and the public. While the review seems to be in some respects just, it goes too far in intimating that Prof. March occupied a position so much coveted, that intentional mistakes were made in his care, with the view of causing a vacancy.

It would appear almost certain that mistakes were made, but it could not be possible that it was so by evil design. It is probable that there are many facts and circumstances which if understood would materially *alter,* we hope improve, the present appearance of the case.

---

*The Baltimore Medical Journal.*

The first number of this monthly publication is published this month. Drs. E. Lloyd Howard and T. S. Latimer are the editors, and it is their aim thus to afford a medium of communication with the medical world to the profession of its vicinity and of the South, The editorial introductory is energetic and high-toned, and the general character of the journal is such as to attract support.

*A Treatise on Intra-Ocular Tumors*, By H. KNAPP, M. D. Translated from the German, by S. COLE, M. D.

We have presented in this work a very minute description of the various Intra-Ocular Tumors which have been observed, mainly of a malignant character. The author regards the eye as an especially advantageous field in which to study the minute anatomy and pathology of malignant diseases, and is sanguine in his expectations of being able to throw much light upon that ancient question of pathologists, "Do tumors which we term malignant have an innocent primary stage? that is, are they at first local affections which afterwards infect the whole organism, or are their first germs always the products of a (tumor forming) dyscrasy previously present in the system?"

The treatment proposed for all forms of malignant disease of the eye, is early and complete extirpation. It is recommended to make careful examination of the optic nerve, and if any appearance of disease can be detected, after enucleation, to excise a still larger piece of the optic nerve from the orbital cavity, drawing it forward with broad forceps and dividing it as far back as possible.

The careful clinical observation upon which the conclusions of the author are based, give much force and value to the teachings of the work, and we believe that all students of Ophthalmology will be deeply interested in it. Pathologists generally, will receive gladly any contributions to the early symptoms and characters of malignant diseases.

---

*Medical Journals and Periodicals.*

"The American Practitioner" is the title of a journal of excellent appearance and character, the first number of which comes to hand this month. Its editors are Professors Parvin & Yandell, the former of whom our readers will remember, has recently removed from Indianapolis to Louisville. The Western Journal of Medicine which Prof. Parvin published when in the former city is now succeeded by the "American Practitioner" published in Louisville. The names of the editors are sufficient guarantee as to the excellence of the journal, which we think is still an improvement upon its predecessor. Original Communications by Drs. Austin Flint, F. J. Bumstead, S. G. Armor and G. C Blackman, are given in the present number, and the aim of the editors is to make this publication a first-class journal, devoted *exclusively to practical Medicine and Surgery*."——The Canada Health Journal, edited by U. T. Campbell, M. D., of London, Ontario, has been commenced this month, and is devoted to the exposition of the laws of Hygiene as relates to public health. The number of contributors and supporters is large, and the Journal, which is published monthly, is furnished at a price such as to secure abundant patronage.——The publishers of the Pacific Medical Journal and the parties interested in the California Medical Gazette, seem to have conspired to "close up business" for editors of the former publication, by means of a so-called sale of the subscription list of the Pacific Medical Journal by its publishers, with whom it had been entrusted, to the California Medical Gazette, and by sending a circular to the subscribers of the Pacific Medical Journal notifying them of its discontinuance, and that they would henceforth be supplied with the Medical Gazette. The editors of the Pacific Journal, as soon as the unhandsome trick was discovered, immediately re-assumed entire control of their Journal, and in absence of a subscription list, give the January number a general distribution among California physicians, whom we are confident will see that the Journal, which is eminently worthy support, suffers no loss by the knavish proceeding.——Hall's Journal of Health for January comes to us greatly transformed in appearance. It is now an illustrated newspaper quarto, has, in addition to Hygienic articles, a literary department, and in its enlarged and improved form, altogether retains its well known vivacity, earnestness and humor.——A copy of the *Revue des Cours Scientifique de la France et de L'Estranger* sent to us, contains an article by Dr. J. H. Salisbury, of Cleveland, Ohio, on the "Causes of Intermittent and Remittent Fevers."

---

### *Scientific and Literary Publications.*

The "Atlantic" for February is an interesting number, and contains an article entitled "Quaff" which considers the phenomena of hallucination, delusions and mania.——The American Educational Monthly which we have regularly received, is the leader of its class of publications, and presents many interesting as well as learned articles.——The American Booksellers Guide, published monthly by the American News Company, is almost indispensable to those who wish to keep posted as to the book publishers market. It not only thoroughly reviews the publishing business of the United States, but contains letters giving full reports of the same in England, France and Germany.——The first number of the Albany Law Journal, a weekly periodical, devoted to the interests of the legal profession, and edited by Isaac Grant Thompson, is received. It would appear from the high literary character of its articles and the ability evinced in them, that the publication must become a favorite with the profession it represents.——Hitchcock's New Monthly Magazine, we can truthfully say, quite surpasses any similar publication that we have seen in the value of the music it presents, the character of its articles and the truthfulness of its criticism. The publisher is well known throughout the country.——Woods' Household Magazine, a popular monthly magazine has a large variety of departments, and is filled with lively and amusing articles. Each number contains forty-four pages, is well worth the dollar subscription, and is published by S. S. Wood, Newburgh, N. Y.——The Scientific American is remarkable for the beauty of its typography, the excellence of its illustrations and the real worth of its articles. It is of first class character, and is held in great favor.——The Educational Bulletin, published by A. S. Barnes & Co., aims to keep the public thoroughly posted as to new educational, classical and school text books, and is of interest to teachers.——The Public Ledger Almanac for 1870, published by G. W. Childs, gives a large amount of information respecting the city of Philadelphia, and is otherwise replete with valuable general intelligence. ——The Old Franklin Almanac for 1870, published by A. Winch, Philadelphia, contains astronomical calculations, a great variety of statistics, chronological tables and useful matter, and is highly valued by its established friends.——Vick's Illustrated Catalogue and Floral Guide has a beautiful appearance and is a credit to its author, Jas. Vick, Rochester, N. Y.——The Printers Bulletin has been again received, and is of interest as bringing to notice the new accomplishments in that art.——Prang's Chromo is an illustrated descriptive guide to the purchaser, of the pieces he so admirably reproduces in chromo.

---

### *Annual Report of the Surgeon General of the United States Army,* 1869.

The health of the army during the past year shows a material improvement over that of the previous one. The Armv Medical Museum has been increased by over two thousand specimens and has been visited by over twenty thousand persons during the year. The manuscript and illustrations of the Medical and Surgical History of the War are in readiness, and the work is progressing toward publication as fast as accuracy will allow. The report on excisions of the head of the Femur, published recently, we have already brought to notice. The number of commissioned medical officers in the army is one hundred and sixty-eight, which is an average of one medical officer to two hundred and four men. There are now two vacancies of Surgeons, and forty-two of assistant Surgeons, and to remedy the deficiency it was necessary (on account of an Act of Congress forbidding appointments or promotions after March 3d, 1869,) to employ physicians under contract· In conclusion Dr. Barnes says :

" The experience of the past three years has shown that the present organization of the medical staff of the army is the best possible for the interests of the service, and that even were all the vacancies now existing, filled, it would be barely adequate in numbers to the demands of our peace establishment. In the British service where troops are always massed in far larger bodies than in ours, the proportion is one medical officer to one hundred and twenty men, and in both the British and Prussian service the relative rank of the medical officers is greater. In Prussia and Austria the adoption of the organization of the medical corps of the U. S. Army, is strongly urged and partially effected. Regimental Surgeons and Assistant Surgeons are to be done away with, and the entire medical corps will be a staff corps, its officers assigned to their duties, irrespective of regiment, by the Minister of War through the Surgeon General."

---

### *Clinical Instruction in Insanity and Diseases of the Nervous System.*

The students of the Medical Class of the University of Buffalo, were, by invitation, favored with a lecture on the 27th inst., from Dr. J. P. Gray, Superintendent of the State Lunatic Asylum for the Insane, at Utica, upon the above mentioned subject. In his remarks, which were quite interesting and instructive, he stated that the success of the late Dr. Griesinger's efforts in instituting Clinics in diseases of the nervous system, at Zurich, Switzerland. and afterwards elsewhere, had been clearly demonstrated and that such a system of instruction was now in operation in the cities of Germany, and was about to be instituted in Scotland, while Dr. Hammond has resumed the same in New York. Very few can give reason why such a course of instruction should not be instituted generally. It has been said that insanity is a disease of the mind, but *there is no mental disease. In-*

*sanity is a disease of the brain, having physical causation, and is therefore a proper subject of medical study.* Physicians who treat *mania a potu*, do not treat the delirium as such, but the pathological condition which occasions it. The same again in delirium accompanying chorea. Another objection to giving attention to this department may be that the treatment of insane is, and is to be confined to the few physicians of the hospitals for the insane. It is a fact that from three to five hundred cases of insanity which require treatment, arise in this State every year ; many of these cases are treated successfully, and many more might be treated successfully if the early symptoms were taken advantage of. Again, diseases of the nervous system have a variety of recognizable symptoms expressive of the stage of the disorder. In acute cases of insanity there are physiognomic signs which are indescribable, but are as marked as those of idiocy and as patent to the clinical observer. Delusion is a state that must be defined in order to distinguish nervous diseases from insanity. Insanity is not a loss of consciousness. The insane man knows that he is insane though he will rarely admit the fact, but he may be convinced of it by reasoning with him, (by asking him if swearing, indifference to family, etc., are characteristic of himself,) and this is one of the therapeutic influences; you are to use his delusion to keep him quiet, but are to treat him as if he had no delusion. In all cases you treat the physical cause of the disturbance, defective nutrition and assimilation, tuberculosis, Bright's disease, or whatever it may be. The study of insanity is entitled to have removed from it its psychological mystery, and as a physical disorder is not incomprehensible to the common practitioner, who can avert in the early stage the serious consequences that otherwise will follow How then can a student be fitted for his profession who is ignorant of the causes, manifestation and treatment of the gravest of diseases. Few cases run their course in a few weeks, and I apprehend the greatest difficulty in clinical teaching will be on account of the chronicity of the affection. A class of typical cases should be examined together. As to the effect upon the patients of taking students through the Hospital, I had observed that it had a good influence upon them, when subsequent correspondence with Prof. Griesinger assured me that such was his own conviction. In New York State the Asylums are all remote from Colleges, and the matter of clinical instruction was considered by the Commissioners in locating the new Asylum in this city, where it will afford unprecedented opportunities which I anticipate will ere long be fully enjoyed by the Institution of which you are members.

